# Tissue-like cultured fish fillets through a synthetic food pipeline

**DOI:** 10.1038/s41538-023-00194-2

**Published:** 2023-05-06

**Authors:** Enbo Xu, Ruihao Niu, Jihui Lao, Shengliang Zhang, Jie Li, Yiyuan Zhu, Huimin Shi, Qingqing Zhu, Yijian Chen, Yuyan Jiang, Wenjun Wang, Jun Yin, Qihe Chen, Xiao Huang, Jun Chen, Donghong Liu

**Affiliations:** 1 College of Biosystems Engineering and Food Science, National-Local Joint Engineering Laboratory of Intelligent Food Technology and Equipment, Zhejiang Key Laboratory for Agro-Food Processing, Integrated Research Base of Southern Fruit and Vegetable Preservation Technology, Fuli Institute of Food Science, Zhejiang University, Hangzhou, 310058 China; 2 Innovation Center of Yangtze River Delta, Zhejiang University, Jiaxing, 314102 China; 3 State Key Laboratory of Fluid Power and Mechatronic Systems, Zhejiang University, Hangzhou, 310028 China; 4 College of Life Sciences, Zhejiang University, Hangzhou, 310058 China; 5 Key Laboratory of 3D Printing Process and Equipment of Zhejiang Province, School of Mechanical Engineering, Zhejiang University, Hangzhou, 310058 China; 6 Key Laboratory for Cell and Gene Engineering of Zhejiang Province, Department of Ophthalmology, Sir Run Run Shaw Hospital, Zhejiang University School of Medicine, Key Laboratory for Corneal Diseases Research of Zhejiang Province, Hangzhou, 310058 China; 7 MOE Key Laboratory of Biosystems Homeostasis & Protection and Innovation Center for Cell Signaling Network, Zhejiang University, Hangzhou, 310058 China; 8 Cancer Center, Zhejiang University, Hangzhou, China

**Keywords:** Cell biology, Biological techniques

## Abstract

Tissue-like cultured meats of some livestock have successfully been established by different approaches. However, the production of a structure similar to fish fillets is still challenging. Here, we develop tissue-like cultured fish fillets by assembly of large yellow croaker muscle fibers and adipocytes with 3D-printed gel. Inhibition of Tgf-β and Notch signals significantly promoted myogenic differentiation of piscine satellite cells (PSCs). The mixture of fish gelatin and sodium alginate combined with a p53 inhibitor and a Yap activator supported PSC viability and proliferation. Based on the texture of fish muscle tissue, a 3D scaffold was constructed by gelatin-based gel mixed with PSCs. After proliferation and differentiation, the muscle scaffold was filled with cultured piscine adipocytes. Finally, tissue-like fish fillets with 20 × 12 × 4 mm were formed, consisting of 5.67 × 10^7^ muscles and 4.02 × 10^7^ adipocytes. The biomanufacture of tissue-like cultured fish fillet here could be a promising technology to customize meat production with high fidelity.

## Introduction

In recent years, based on the concerns about efficiency, sustainability, environmental burden, and animal welfare, cell culture meat technology has emerged as an alternative to partially replace the traditional livestock industry in meat production^1,2^. Tissue-like cell-cultured meat with a composition and a structure similar to real muscle tissue, comprising mostly adipocytes, and aligned muscle cells. With the rise of digital modeling technology, several researchers have successfully constructed meat tissues of livestock such as cow^3^ and pig^4^ using 3D bioprinting technology. The in vitro three-dimension (3D) cell culture conditions including culture medium (for instance fetal bovine serum) and supporting materials benefit from the studies of human cell or other mammalian cell cultures with the aims for regenerative medicine and organoid generation^5,6^. However, the primary purpose, cost and scale of food construction and other key aspects (e.g. target species, nutrients, and organoleptic characteristics) are totally different from those of biomedical tissues^7^. For the cell-cultured meats, establishing their own approaches still faces many challenges. One of the challenges is to construct the scaffold with efficient and edible extracellular matrix (ECM), or degradable supporting materials, which allow cells with high adaptability in protrusion, adhesion, translocation, proliferation, differentiation, and fusion^2,8^.

Seafood is favorite food for many people because of its taste and rich in various proteins, omega-3 fatty acids, and micronutrients. The increase of population, coupled with environmental stress and climate-change, has led to the overexploitation of marine food resources in recent decades, which has had enormous impacts on ecosystem^9^. Therefore, many aspects, such as scientific assessment and policy management, as well as innovative technologies (for instance: cell-cultured fish meat), are urgent for the sustainability of seafood production. However, unlike livestock, few studies have used marine fish to explore in vitro myogenesis. There is great diversity of muscle types in marine fish, which vary in physiological and metabolic status. At present, to find out what type of species, cells, culture conditions and supporting materials can be used for the production of tissue-like cell-cultured meat in marine are the major challenges. A recent study demonstrated a potential to develop fish meat from fin-derived fibroblast cells^10^. The fibroblast cells were isolated from thread-sail filefish (*Stephanolepis cirrhifer*) fins. By changing sera or culture media, the fibroblast cells were induced to differentiate into various cell types such as: muscle cells, adipocytes, and neurofilaments, etc. A sashimi-like fish meat was produced by multiple-layered cell sheet culture. As the first enterprise in Europe to produce cell-cultured fish, the Bluu Seafood company in Germany has claimed to successfully prepare fish meat strips and pellets using *Oncorhynchus* cells^11^. Due to the lack of fish muscle tissue textural properties and a 3D scaffold for mechanical support, production of tissue-like cultured fish fillets is still an unfinished task.

Large yellow croaker (*Larimichthys crocea*), a warm-temperate migratory fish and famous marine economic fish species in Eastern Asia, is favored by consumers because of its delicious flavors and richness in nutrients such as unsaturated fatty acids. However, the wild resources of large yellow croaker are seriously exhausted due to overfishing. So far, only few studies have focused on the textures of fish muscle tissues and supporting materials of scaffold for fish cell adhesion, proliferation, differentiation, and fusion from 2D to 3D culture. In this report, we isolate Pax7^+^/Myf5^−^ piscine satellite cells (PSCs) and piscine adipose-derived stem cells (PADSCs) from large yellow croaker to develop tissue-like cultured fish fillets by an approach based on 3D-printed gelatin-based gel.

## Results

### Isolation, proliferation, and differentiation for the PSCs and PADSCs

Most of the muscles of large yellow croaker belong to the white muscle of vertebrates. It is mainly composed of skeletal muscle and fat. Muscle satellite cells are considered as the main stem cells and are crucial for muscle growth and regeneration. They are generally quiescent until to be activated and become myogenic cells, i.e., myoblasts, which will undergo proliferation and myogenic differentiation to form new muscle fibers.

**Fig. 1:**
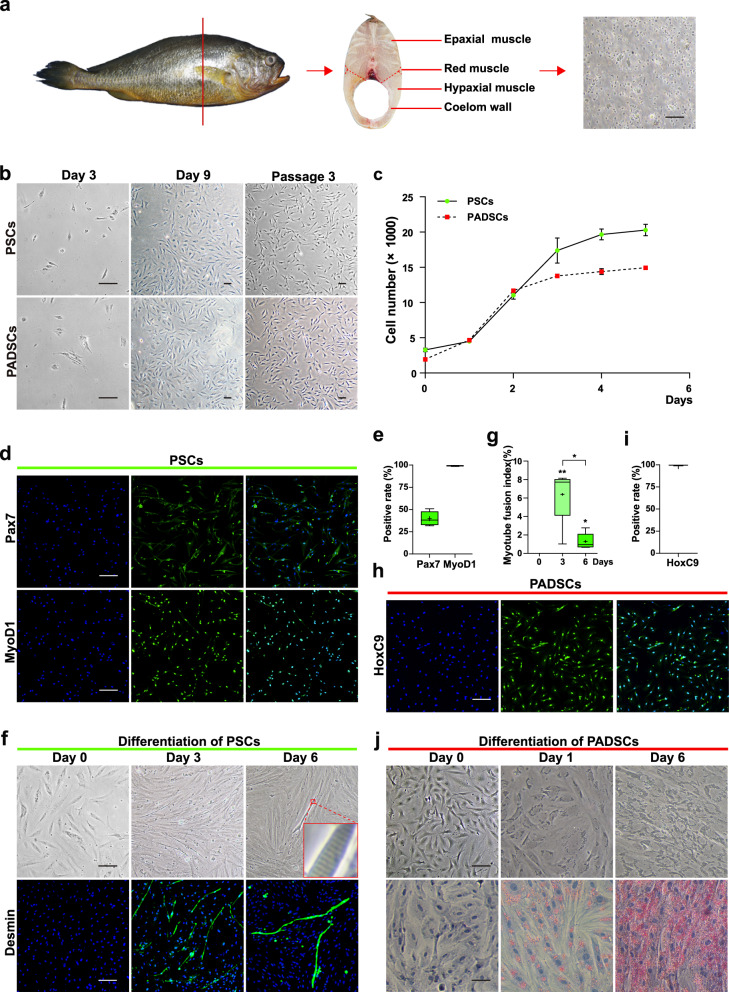
Proliferation and differentiation of large yellow croaker PSCs and PADSCs. **a** Diagrams showing that epaxial muscles and coelom walls of large yellow croaker were used to isolate the primary culture of PSCs and PADSCs respectively. Scale bars: 80 µm. **b** Primary cultures at different time points and passage 3 of PSCs and PADSCs. Scale bars: 80 µm; **c** The growth curves of PSCs and PADSCs at passage 3 were analyzed by CCK-8 method with three replicates. **d** Representative images of immunofluorescence staining of PSCs at passage 3. Green: Pax7 or MyoD1; Blue: Hoechst 33342 for nuclear staining. Scale bars: 80 µm. **e** Average percentages of Pax7- and MyoD1-positive cells derived from image **d** with five replicates. Box indicates IQR; whisker indicates min or max; plus shows mean. **f** Representative images of bright-field and fluorescence staining of PSCs during myogenic differentiation at day 0, 3, 6. Green: Desmin; Blue: Hoechst 33342; Scale bars: 80 µm. **g** Myotube fusion index counted from image **i** with five replicates. Box: IQR; Whiskers: min or max; Plus: mean. **h** Image of immunofluorescence staining of PADSCs at passage 3. Green: HoxC9; Blue: Hoechst 33342. Scale bars: 80 µm. **i** Average percentage of HoxC9-positive cells counted from image **f** with 5 replicates. Box: IQR; Whisker: min or max; Plus: mean. **j** Representative images of bright-field and oil red O staining of PADSCs during adipogenic differentiation at day 0, 1, 6. Lipid droplets in red were stained by oil red O and nuclei in blue were stained by hematoxylin. Scale bars: 40 µm.

The culture of large yellow croaker muscle cells has been reported recently, where tissue block culture was used^12^. To isolate more pure muscle stem cells, we followed an enzymolysis approach from higher vertebrates and other fishes^13^. The epaxial muscle of large yellow croaker was physically decomposed. The satellite cells were released by collagenase and trypsin treatments and collected by filtration (Fig. 1a). By optimizing the combination of different media, serum ratio, and growth factors (data not shown), the proliferation medium of PSCs was finally determined as high-glucose DMEM with 15% fetal bovine serum (FBS) and 10 ng/ml bFGF. At 9 days post-incubation, the primary cultured cells reached about 80% confluence, and the cell morphology began to appear as fibroblasts. After 3 passages, these cells gradually displayed typical fibroblasts morphology (Fig. 1b). Cells from Passage 3 were used to evaluate their proliferation ability with CCK8 assay kit. The data showed that the number of cells doubled less than 2 days after seeding, and the proliferation plateau began to appear on the third day (Fig. 1c). This proliferation capacity was maintained for at least 26 passages (data not shown here). The cells from Passage 3 were also characterized by immunofluorescent staining with the specific myoblast markers such as Pax7 and MyoD1. The results showed that about 43% of the cells were Pax7 positive, and 99% of the cells were MyoD1 positive (Fig. 1d, e). These data suggested that the isolated cells possessed the properties of PSCs, which are MyoD1 positive myoblasts after 3 passages.

To evaluate the differentiation potentials of these PSCs, even after multiple passages, the serum starvation myogenic differentiation protocol for mammalian satellite cells was initially adopted. Although myotubes were induced within 2–3 days with medium containing 2% horse serum (HS), they detached shortly and died soon after. To improve differentiation efficiency and survival rate, different combinations of basal medium, serum ratios, and myogenic factors were evaluated. Finally, the F12 medium containing 8% HS, 10 ng/ml IGF-1, 50 nM necrosulfonamide and 200 μM ascorbic acid was identified as an effective recipe for myogenic differentiation. On day 3 of culture in this differentiation medium, elongated myotubes began to appear, and on day 6, striations similar to skeletal muscle were observed in some of myotubes (Fig. 1f). Immunofluorescent staining of Desmin (a muscle-specific protein) showed that Desmin-positive myotubes with multiple-nuclei were detected on both days 3 and 6 (Fig. 1f, g). For unexpected reasons, the fusion index (the number of nuclei in Desmin-positive multinucleated myotubes was divided by the total number of nuclei in a certain area) was significantly higher on day 3 (6.39%) than on day 6 (1.30%) (Fig. 1g). Although the average number of nuclei in individual myotubes was higher on day 6, the total number of myotubes on day 6 was much less than on day 3 (Fig. 1f).

The isolation, proliferation, and differentiation of large yellow croaker preadipocytes have already been reported^14^. We followed the protocol to isolate PADSCs from ventral coelomic fat tissues. Similar to PSCs, the culture conditions for PADSC proliferation and differentiation were optimized. The proliferation medium was high-glucose DMEM containing 8% FBS, which was reduced by half compared with that previously reported^14^. The cell growth rate maintained high in the first two days and reached a plateau after three days (Fig. 1c). Immunofluorescence staining of adipose tissue marker HoxC9 showed that almost all cultured cells were positive (Fig. 1i), suggesting that these cells originated from abdomen fat tissue were PADSCs. The feature of PADSCs was confirmed by the adipogenic differentiation. DMEM/F12 containing 10% HS, 10 μg/mL insulin, 0.5 μm IBMX, 0.25 μm dexamethasone, and 1% Lipid Mixture Solution, was used as the differentiation medium. Staining with Oil Red O showed that lipid droplets began to appear in almost all cultured cells on day 1, and the number and size of lipid droplets increased obviously on day 6 (Fig. 1j). These results demonstrated that the isolated cells were PADSCs.

### Improving the myogenic differentiation efficiency by transcriptomic analysis

**Fig. 2:**
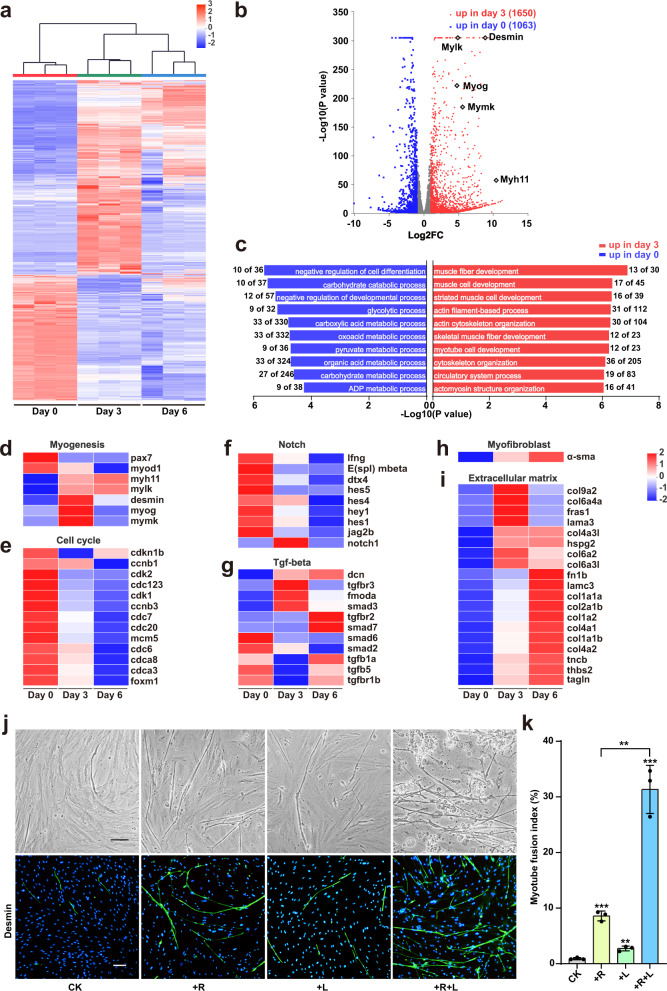
Improving the myogenic differentiation efficiency by transcriptomic analysis. **a** Heatmap showing the z value of all differentially expressed genes during myogenic differentiation between day 0, day 3 and day 6. Genes (rows) and sample (columns) were clustered using average linkage clustering with Euclidean distances. **b** Volcano plot showing differentially expressed genes between day 0 and day 3. Significantly differentially expressed myogenesis genes were highlighted. **c** Top 10 enrichment GO analysis for up-regulation (red bar) and downregulated (blue bar) DEGs at day 3, compared to day 0. The number indicated the proportion of the significantly up/downregulated genes in total genes per gene ontology. **d**–**i**, Heatmaps showing the expression patterns of genes corresponding to myogenesis (**d**), cell cycle (**e**), notch signal pathways (**f**), TGF-beta signal pathways (**g**), myofibroblastic (**h**), extracellular matrix (**i**), from day 0 to day 6. **j** Representative bright-field and immunofluorescence images of PSCs at day 6 in different differentiation media. In first two days, the cells were cultured with F12 medium containing 8% HS, 1×PS and with or without Repsox (R, 5 μM) and LY411575 (L, 10 nM). After 2 days. The culture was added with same volume DMEM medium containing 8% FBS, 200 nM dexamethasone, 1×PS. Scale bars: 80 µm. **k** Myotube fusion index counted from images **j** with three replicates. Statistical analysis was performed on relevant data using Student’s two-tailed *t*-test. Error bars indicated s.d. **P < 0.005, ***P < 0.0005.

In addition to the low efficiency of myogenic differentiation, another major concern in our PSC differentiation culture was the decrease of the fusion index from day 3 to day 6 (Fig. 1g). To address these questions, we performed transcriptomic profiling by bulk RNA-seq at different time points (day 0, 3, 6) of differentiation. Clustering analysis showed that the expression profile changes on day 3 were more dramatical than those on day 6, compared with day 0 (Fig. 2a), which was consistent with the fusion index. The differentially expressed genes (DEGs) analysis showed that 1,650 of 22,093 genes were significantly upregulated (log2 FC > 1, FDR < 0.05), and 1063 were significantly downregulated (log2 FC < −1, FDR < 0.05) from day 0 to day 3 (Fig. 2b). Gene ontology (GO) enrichment revealed that the upregulated genes were predominantly related to terms associated with muscle development and function, whereas most of the downregulated genes were enriched under terms such as negative regulation of cell differentiation, developmental process and some metabolic process, suggesting that serum starvation induced myogenic differentiation (Fig. 2c). However, we noted that genes related to cell cycle regulation were not in the downregulated gene category, which was different from that previously described in mice and bovine^15^. There were at least two reasons for phenomenon. The first one was the low frequency of myogenic differentiation of PSCs. The second reason was the low viability of fish myocytes in the medium lacking serum (data not shown).

The results of DEGs from comparison showed that 664 genes were significantly upregulated, and 1165 were significantly downregulated between day 6 and day 3 (Supplementary Fig. S1a), wherein the term of mitotic cell cycle process was also not enriched into the downregulated gene category (Supplementary Fig. S2a). While, the term of mitotic cell cycle process was enriched into the downregulated gene category form DEGs between day 6 and day 0 (Supplementary Fig. S2b), where 1636 genes were significantly upregulated, and 1428 were significantly downregulated (Supplementary Fig. S1b). These data indicated that cell proliferation gradually decreased during the differentiation from day 0 to day 6 (Fig. 2e).

Gene expression profiles of myogenic factors and differentiation marker genes showed that the myogenic stem/progenitor marker *pax7* and the early myogenic determination marker *myod1* were continuously downregulated from day 0 to day 6 of differentiation; the late myogenic determination marker *myog*, the myogenic differentiation markers *desmin* and the myoblast fusion factor *mymk* were upregulated on day 3 and then downregulated on day 6; the skeletal-muscle-specific markers *myh11* and *mylk* steadily increased from day 0 to day 6 (Fig. 2d, Supplementary Fig. S1c). Interestingly, the myogenic regulator *myf5* was not detected in the whole process. This might be because the PSCs isolated from epaxial muscle of large yellow croaker were Myf5^−^. The vertebrate epaxial muscle originates from embryonic dermomyotome, in which there is developmental heterogeneity of the cell lineage. At least two populations of myoblast progenitors coexist in developing dermomyotome. The major population is the fast-cycling population, Pax7^+^/Myf5^+^, which possesses limited differentiation capacity and will participate in terminal differentiation more quickly^16^. The minor population is the slow-cycling population, Pax7^+^/Myf5^−^, with self-renew capability, which is important to maintain muscle homeostasis and regeneration^17–19^. Our results demonstrated that the PSCs isolated from fish epaxial muscle belong to Pax7^+^/Myf5^-^ slow-cycling population.

In order to find out what signals might be responsible for low myogenic efficiency, we performed KEGG analysis. The results showed that a number of important signaling pathways, including MAPK, TGF-β, Wnt, Notch, hippo, HIF-1, etc., were enriched in the DEGs (Supplementary Fig. S1c). Among them, two signaling pathways, Notch and TGF-β, attracted our attention in particular. In muscle tissue, Notch signaling is required for the maintenance of quiescence of satellite cells^20^. TGF-β signaling plays a negative role in myogenesis of adult muscle tissue (reviewed by Burks & Cohn^21^). Balance between Notch and TGF-β signaling regulates proliferation of satellite-cells, and attenuation of TGF-β restores regeneration in old, injured muscle^22^. In the differentiation process of PSCs, the expression of main members of Notch pathway on day 3 such as *lfng, hes4, hey1* and *hes1*, did not significantly decrease, and the expression of *notch1* even increased, though all of these genes decreased on day 6 (Fig. 2f). Since the downregulation of Notch signaling at the early stage is crucial for PSCs to transition from stem cell fate into differentiation state, relatively high level of Notch signal on day 3 might be one of obstacles for myogenic differentiation.

The changes of TGF-β signaling were more complicated in myogenic differentiation of PSCs (Fig. 2g). Compared to day 0, on day 3, the expression of some members of TGF-β signaling (*dcn, tgffbr3, tmoda*, and *smad3*) increased, while the expression of some members (*smad6, smad2, tgfb1a, tgfb5*, and *tgfbr1b*) decreased; on day 6, the expression of *dcn*, *tgfbr2*, and *smad7* increased, while the expression of *smad6* and *smad2* decreased (Fig. 2g). The data indicated that TGF-β signaling was dynamically changed during the induction of myogenic differentiation. A previous study reported that TGF-β induced myogenic cells to transform into myofibroblastic cells after injury^23^. Therefore, we wondered whether the low fusion index was due to TGF-β signal induced transformation of PSCs into myofibroblasts in the serum starvation culture. The expression of α-smooth muscle actin (*α-sma*) (a specific marker for myofibroblast) and most extracellular-matrix (ECM) components was upregulated on day 3 and upregulated further on day 6 (Fig. 2h, i), indicating that myofibroblastic differentiation occurred in the serum starvation culture.

To improve the myogenic efficiency and prevent myofibrosis, we inhibited Notch and TGF-β signals with two small molecule drugs: LY411575 (a Notch inhibitor) and RepSox (a TGFβR-1/ALK5 inhibitor) respectively. Two chemicals were added to the basal medium (F12 medium containing 8%HS, 1×PS) for the culture of first two days and DMEM medium (containing 8% FBS, 200 nM dexamethasone and 1×PS) for the culture of later stage. On day 6, the numbers of myotubes were increased slightly in the treatments with either LY411575 or RepSox alone, and increased much obviously in the treatment with both chemicals together (Fig. 2k). The fusion index in the control was about 1%, which was increased to 8% and 3.5% by the treatment of RepSox or LY411575 respectively, and synergistically increased to 32% by the treatment of two chemicals together (Fig. 2k). The results demonstrated that the myogenic efficiency of PSCs was greatly improved by inhibition of Notch and TGF-β signals.

### Effects of supporting materials of 3D-scaffold on fish cell adhesion and growth

**Fig. 3:**
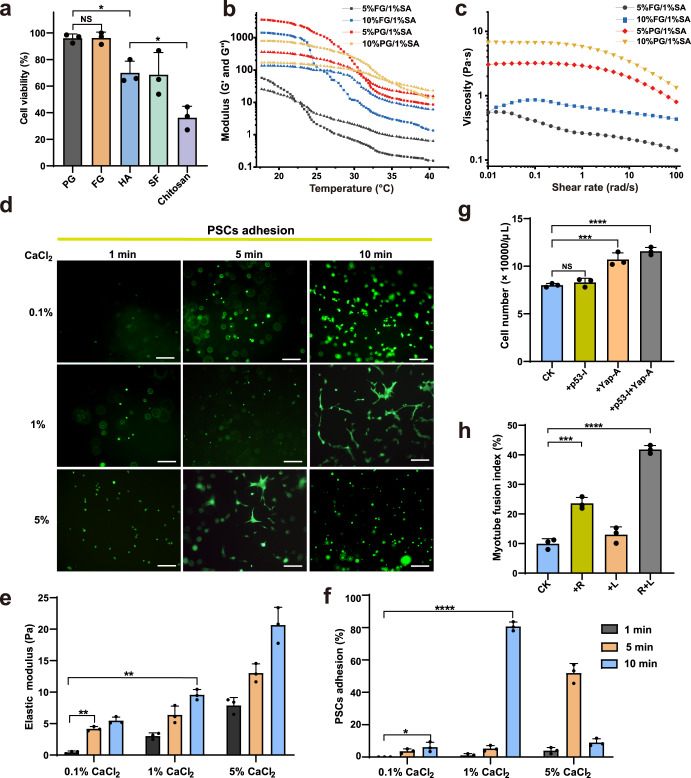
Effects of supporting materials of 3D-scaffold on fish cell adhesion and growth. **a** Average cell viabilities in different supporting materials including porcine gelatin (PG), fish gelatin (FG), Hyaluronic acid (HA), silk fibroin (SF), and chitosan. Statistical analysis was performed on relevant data using Student’s two-tailed *t*-test. Error bars for three replicates indicated s.d. NS: not significant; *P < 0.05. **b**, **c** Gel point (**b**) and viscosity (**c**) of supporting materials with different ratios of gelatin basis (5/10%) and 1% sodium alginate (SA). The square and triangle symbols in **b** represented G’ and G” respectively. **d** Calcein-AM fluorescence images of PSCs treated with different concentrations of CaCl_2_ for different times. The non-adherent PSCs were spherical, while the adherent PSCs became spindle-shaped and gradually underwent proliferation. Scale bar, 50 μm. **e**, **f** Elastic modulus of scaffolds (**e**) and cell adhesion rates of PSCs (**f)** treated with different concentrations of CaCl_2_ for different times. The experiments were repeated for three times and statistical analysis was performed on relevant data using Student’s two-tailed *t*-test. Error bars indicated s.d. **P < 0.005, ****P < 0.0001. **g** Average number of PSCs measured with CCK-8 kit at day 5 of 3D culture with three replicates. Error bars indicated s.d. **P < 0.005, ***P < 0.0005, ****P < 0.0001. **h** Average myotube fusion index in different 3D Culture Conditions as indicated. R: Repsox; L: LY411575. Statistical analysis was performed on relevant data using Student’s two-tailed *t*-test. Error bars for three replicates indicated s.d. **P < 0.005, ***P < 0.0005.

To select the supporting materials for 3D-culture of PSCs, we compared various materials from different resources including porcine and fish gelatins (PG/FG)^24^, hyaluronic acid (HA)^25^, silk fibroin (SF)^26^, and chitosan^27^. The evaluation of material-to-cell compatibility showed that gelatin-based gel had the highest cell viabilities (Fig. 3a and Supplementary Fig. S3). As a partial hydrolysate of collagen, gelatin has natural biocompatibility and can provide an extracellular microenvironment suitable for the survival of fish cells^28^. The best temperature for fish cell growth is about 27 °C^29^. To maintain the proliferation capacity of fish cells, the gel point of 3D culture materials for large yellow croaker cells should be lower than ~27 °C, which is different from conventional bio-inks for human or other mammalian cells (about 37 °C). Therefore, we made bioinks by using porcine- and fish-derived gelatin combined with sodium alginate (SA) to measure their rheological properties. The gelation temperature of FG material was lower than that of PG material. The gel points (i.e. G’ = G”) of 5% and 10% FG were 22.15 and 25.51 °C, respectively (Fig. 3b). PG inherently contained higher amounts of hydrophobic amino acids and forms stronger hydrophobic interactions than FG^24^, resulting in higher gel points (31.25 and 34.39 °C for 5% and 10% PG, respectively) which could not be used for fish cell culture. Both PG and FG materials exhibited shear thinning behavior at shear rates from 0.1 to 100 rad/s. It was known from Supplementary Fig. S3b that FG had a lower contact angle than PG, indicating its better hydrophilicity.

Subsequently, the gel (10% FG/1% SA) were mixed with PSCs and treated with CaCl_2_ at different concentrations for different times to pre-fabricate scaffold materials with altering stiffness of elastic modulus. We found that 1% CaCl_2_ solidification for 10 min or 5% CaCl_2_ solidification for 5 min induced most PSCs to adhere (Fig. 3d). It is known that large changes in matrix stiffness influence the focal-adhesion and cytoskeleton structures for differentiated cells^30,31^. Mechanosensing receptors on the PSC membrane activated actomyosin-mediated contraction and cytoskeletal rearrangement after sensing matrix stiffness in a narrow range of ~9–14 kPa (Fig. 3e, f). The cell adhesion rates could reach up to 80.69% for the PSCs treated with 1% CaCl_2_ for 10 min (Fig. 3f). The activated PSCs became spindle-shaped and gradually proliferated, migrated and differentiated in the 3D culture materials, while non adherent cells were unable to formation subsequent muscle tissue.

We observed that PSCs had low cell proliferation rate in the 3D culture environment. To deal with this problem, a p53 (a well-known tumor repressor) inhibitor (Pifithrin-α) and a Yap (a positive regulator in cell survival and proliferation) activator (XMU-MP-1) were applied in the culture. The results showed that the cell density in the control medium was about 8.02 × 10^4^ cells/μL, which was increased to 8.31×, 10.73× and 11.57 × 10^4^ cells/μL by the treatment of Pifithrin or XMU-MP-1 or two chemicals together (Fig. 3g). The data demonstrated that the cell proliferation rate of PSCs was significantly improved by the combination of two chemicals.

Myogenic differentiation experiments showed that the fusion index in the 3D culture with the basal medium (F12 medium containing 8%HS, 1×PS) was 9.94%, which was much higher than that (about 0.91%) in 2D culture, suggesting that cells in 3D culture inherently have a higher fibrogenic tendency than cells in 2D culture due to the stimulation of supporting material stiffness. Consistent with 2D culture, combination of LY411575 (Notch inhibitor) and RepSox (TGFβR-1/ALK5 inhibitor) also significantly increased the fusion index in 3D differentiation culture (from 9.94% to 41.79% on day 7) (Figs. 2k and 3h).

### Muscle tissue formation under guidance of biomimetic model of scaffold

**Fig. 4:**
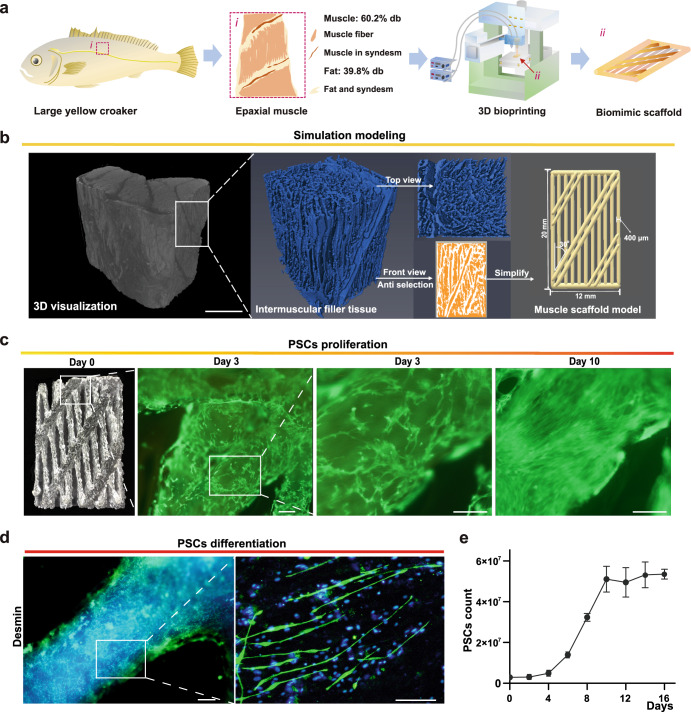
Muscle tissue formation under guidance of biomimetic model of scaffold. **a** Schematic diagram of construction of biomimetic 3D-printed model based on native large yellow croaker muscle tissue. **b** Micro-CT images of native fish epaxial muscle. Blue: Intermuscular filler tissues; Orange: Muscle tissues; Muscle scaffold model was designed from simplified native muscle texture. Scale bar: 1 mm. **c** Calcein-AM fluorescence images of PSCs in scaffolds at day 3 and 10 days of proliferation. Scale bar, 100 μm. **d** Representative fluorescence image of myobubes in 3D culture at days 7 of differentiation. The PSCs in scaffolds were cultured in proliferation medium for 10 days, and then transferred to differentiation medium for 7 days. Green: Desmin; Blue: DAPI. Scale bar, 100 μm. **e** Growth curve of PSCs in optimized gelatin-based scaffold. Error bars for three replicates indicate s.d.

The reconstruction of the microstructure of cultured meat, especially based on its real biological structure, has been a challenge due to the reproduction of tissue-like texture. In addition to the supporting role, layered porous scaffolds can also enhance cell adhesion and proliferation, which indicates the importance of microstructure to the biological function of scaffolds^32,33^. On the purpose of reconstruction of a printable optimized model of scaffolds, we used a digital analysis of micro-CT scanning and computerized tools to analyze the textures of large yellow croaker muscle tissue, and conducted a 3D bioprinting method to manufacture the biomimic scaffolds (Fig. 4a).

The 3D structure of the micro-CT slice images of large yellow croaker epaxial muscle tissue showed that fish muscle fibers were an unregular polygonal shape and were surrounded by a layer of myocommata composed of collagen and syndesm (Fig. 4b and Supplementary Fig. S4a). Fat tissues as intermuscular filler tissues distributed between muscle fibers (Fig. 4b). 3D volume rendering gave a brand-new view of large yellow croaker muscle compared to conventional imaging (data not shown). Muscle fibers and myocommata seemed to be anisotropic diffusion in space, forming a relatively gradual change in 3D structure. However, for high-precision scaffold construction, it was very difficult to adjust the irregular trajectory and needle diameter for 3D bioprinting of myofibrils^34^. Therefore, in order to simulate muscle fibers, we had to simplify the digital information of fish tissue structure to have a periodic superposition of two-dimensional plane with proper size (see below). The simplified flows were showed in a case of cross section of epaxial muscle tissue (Supplementary Fig. S4b), which guides the definition of longitudinal biomimic scaffolds shown in Fig. 4b. Due to the limitations in methods and equipment of 3D bioprinting for biomimetic and edible cell scaffolds, especially the bionic structure with spatial anisotropy, we designed a regular lattice model based on the calculation of fish micro-CT slice images (Fig. 4b and Supplementary Fig. S4c, d). According to the image processing and numerical transformation^35^, the digital muscle fibers were extracted in parallel or interlaced (with angles of 30°), and labeled as muscle scaffold model (Fig. 4b). The anisotropic lattice structure was rendered blue representing fat and syndesm. The top view of intermuscular filler model was shown in Fig. 4b, whose inverse shape was similar to the microstructure of real large yellow croaker muscle (front view in orange). By this, we obtained the ratio of muscle and fat (~6:4) for modeling.

Then a 3D extrusion-printer was used to construct large yellow croaker muscle scaffold with the gel compound (10% FG/1% SA) mixed PSCs at a concentration of 5 × 10^6^ cells/mL. Each layer of the muscle scaffold consisted of 17 gel fibers, six of which were inclined at 30° to the other gel fibers (Fig. 4b). The printing was repeated ten times to form a muscle model with a length of 20 mm, a width of 12 mm and a thickness of 4 mm. All printing parameters are shown in Supplementary Table S1. The muscle scaffold was manufactured in batch process and placed in the proliferation medium. Cell viability was 86.2% in the printed scaffolds, indicating that the gelatin-based bioink had a good protection for the cells. On day 3, spindle-shaped PSCs were observed without ordered growth direction on biomimic muscle scaffold; On day 10, proliferated PSCs almost filled the muscle scaffold, and then the culture was replaced with differentiation medium (Fig. 4c). On day 7 of differentiation culture, myotubes were formed and integrated into scaffolds with aligned rearrangement (Fig. 4d). These aligned muscle fibers consisted of up to 5.35 × 10^7^ of PSCs, with the cell growth rate maintained high from day 4 to day 10, and reached a plateau after seven days of differentiation (Fig. 4e).

### Characterization of tissue-like cultured fish fillets

**Fig. 5:**
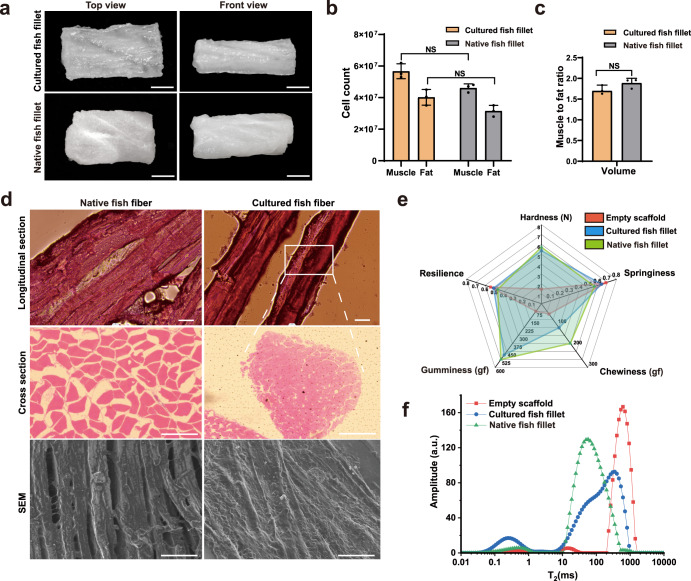
Characterization of tissue-like cultured fish fillets. **a** Representative images of cultured and native tissue fillets of large yellow croaker. Scale bar, 5 mm. **b** Average numbers of muscle cells and adipocytes in cultured fish fillets and native fish fillets with a size about 0.96 cm^3^. Error bars for three replicates indicated s.d.. NS: not significant. *P < 0.05. **c** Muscle and fat ratios in cell cultured fish fillets and native fish fillets. Error bars for three replicates indicated s.d. NS, not significant. *P < 0.05. **d** Representative images of muscle fibers of native fish fillets and cultured fish fillets. Red: hematoxylin-eosin (HE) staining; SEM: scanning electron microscopy; Scale bar, 100 μm. **e** Texture properties of empty scaffold, cultured fish fillet and native fish fillet. **f** Water distribution of transverse relaxation time (T_2_) spectra. “a.u.” denoted arbitrary unit.

The differentiated PADSCs of adipocytes were filled into the muscle scaffolds to form cell-cultured fish fillets (Fig. 5a). To evaluate cultured meats, first, we compared the numbers and proportions of muscle cells and adipocytes in the cultured fish meats and muscle tissues of raw large yellow croaker (Fig. 5b, c). The results showed that the average numbers of muscle cells and adipocytes were respectively 5.67 × 10^7^ and 4.02 × 10^7^ in about 0.96 cm^3^ cultured fish fillet, which were slightly higher than those in the same volume of native muscle tissue (4.61 × 10^7^ and 3.22 × 10^7^, respectively).

Next, we used histological staining and SEM to analyze the microstructure of cultured fish meats. Hematoxylin-eosin and immunofluorescence section staining showed that after 17 days culture, the muscle fibers exhibited a similar arrangement to the native muscle fibers (Fig. 5d and Supplementary Fig. S5a). The muscle fibers in the scaffold were composed of myotubes with a diameter of about 400 μm. The ratio of muscle to fat in cell-cultured fish fillets calculated from the cross-sections was 1.73:1, which was similar to that of native muscle tissue (1.88:1).

Furthermore, we performed textural analysis on empty scaffolds and scaffolds seeded with muscles/adipocytes. Textural measurements showed that the hardness of cultured fish fillet was 5.37 ± 0.42 N, which was in the same range as that of native yellow croaker fillet (5.72 ± 0.80 N) (Fig. 5e). There were also no obvious differences in gumminess, resilience, and springiness between cultured meats and native muscle tissues. The hardness, gumminess, and chewiness were increased, and springiness and resilience were decreased in scaffolds seeded with muscles/adipocytes, compared to empty scaffolds. However, chewiness of cultured fish fillets was lower than that of native muscle tissues. It might be due to the relatively higher free water content in cultured fish fillets (see below).

To explore the distribution and variation of water in cultured fish fillet, we analyzed the mobility and proportion of water molecules by low-field NMR relaxation behavior. By multi-exponential fitting analysis^36^, the cultured meats showed three peaks at 0.25 ± 0.00 ms, 2.83 ± 0.62 ms, and 335.87 ± 26.40 ms, representing T_2b_, T_22_, and T_23_ respectively, which accounted for tightly bound water, immobilized water, and free water (Fig. 5f). Although empty scaffolds and native muscle tissues also had these three peaks as cultured meats, the contents of free water and bound water were different in the three samples. The free water content in cultured meats decreased by 8.23%, while the tightly bound water content increased by 9.52%, compared to those in empty scaffold, indicating that the muscle fibers formed in the cultured fish fillets transformed some of the free water into bound water. However, the water distribution in cultured fish fillets (11.10% tightly bound water, 0.95% immobilized water, and 87.95% free water) still differed from that in native muscle tissue (3.33% tightly bound water, 96.48% immobilized water and 0.19% free water) (Supplementary Fig. S5c). Thus, the simulation of ECM in cultured fish meats could be focused in future work.

Taken together, the appearance and many characteristics (including total cell numbers, ratio of muscle cells and adipocytes, hardness, gumminess, resilience, springiness, etc.) of our cultured fish fillets were similar to those of native muscle tissues of large yellow croaker.

## Discussion

Biomanufacture of textural tissue-like cultured meat requires efficient proliferation and differentiation of isolated cells induced in food grade scaffolds for 3D culture in vitro. Bovine satellite cells^15^, porcine^37^, and rabbit^38^ skeletal muscle myoblasts have successfully been used to develop cultured meats. However, the composition and structure of tissue-like meats similar to native muscle tissues, need an aligned assembly of main ingredients including adipocytes, muscle cells, and supporting materials with a high moisture content^2,3^, which are quite different between species. Although cultured fish meat was exploited by multiple-layered cell sheet culture with fibroblast cells from thread-sail filefish fins^11^, the robust adhesion, proliferation, differentiation, and fusion of piscine stem cells on 3D scaffolds are still a huge challenge. In this report, based on the texture of large yellow croaker muscle tissue, we assembled large yellow croaker muscle fibers and adipocytes with 3D-printed gel to develop tissue-like cultured fish fillets.

The PSCs and PADSCs were separately isolated from epaxial muscles and ventral coelomic fat tissues. These two types of cells had efficient proliferative abilities and were capable to differentiate into myotubes and adipocytes respectively. Although the efficiency for PADSCs to differentiate to adipocytes was very high, the efficiency for PSCs to differentiate to myotubes was extremely low according to the traditional serum starvation induction method. To improve the differentiation efficiency of PSCs, transcriptomic analysis on myogenic differentiation was performed. Very interestingly, we found that the isolated PSCs were Pax7^+^/Myf5^-^. The Pax7^+^/Myf5^-^ PSCs belong to the slow-cycling population with self-renew capability and play an important role in muscle homeostasis and regeneration. Based on the transcriptomic analysis, the efficiency of PDS myogenic differentiation was tremendously improved from 1 to 32% by the combination of two inhibitors respectively for TGF-β and Notch signals.

ECM composition (e.g. RGD, integrin and other functional factors) and stiffness play an important role in cell fate determination^31,39^, which are quite different between species. Therefore, for the production of tissue-like meats, the key step is to find the supporting materials of 3D scaffolds to provide a microenvironment of chemo/mechanosensing for cell proliferation and differentiation^8,30^. Among PG, FG, HAMA, SF and chitosan, we found that gelatins provided good cytocompatibility for PSCs. However, PG is not suitable for 3D bioprinting fish cells due to its high gel point that unable to mix with cells at <27 °C. After pre-extruded of the two concentrations of fish gelatin, the cell viability in 10% FG/1% SA (86.2%) was higher than that in 5% FG/1% SA (80.6%), which was due to the high viscosity system provided more protection to the cells during extrusion. Therefore, we chose 10% FG/1% SA as the bioink formulation for the next study. The PSC adhesions on FG-based gel were enforced by calcium ion at appropriate stiffness. The cell viability in the scaffolds was significantly increased by the combination of a p53-inhibitor and a Yap-activator. Similar to the 2D culture, the myogenic differentiation efficiency of 3D culture was also greatly improved by the two chemicals (inhibitors, respectively for TGF-β and Notch signals). Although these small molecule compounds used in the cell cultures benefit from basic biomedical research, as a consumer food, the safe residual dose in cell-cultured meats should be a highly considered issue. For future studies, natural secondary metabolites with the same efficacy would be a better choice than artificially synthesized small molecule compounds.

Our 3D scaffold was constructed in high throughput based on FG-gelatin gel with a proper gel point (25.51 °C), that is the key for bioprinting and maintaining fish cell viability. In a previous report, the beef steak-like cultured meat was manually assembled one by one using linear tendon-gels^3^. Inspired by this, we designed a mass customization of FG-gelatin scaffold to simulate native fish muscle tissue. The digital analysis on the textural properties of large yellow croaker muscle tissue showed a regularity of muscle fiber and fat distributions (with a ratio of ~6:4), as previously described^40^. We extracted the main information from the epixial muscle of large yellow croaker to simplify printable model of 3D scaffold. After proliferation and differentiation culture, the aligned muscle fibers differentiated from PSCs were observed along with the scaffold.

Analysis on the composition and texture of our cultured fish fillets showed that the numbers of myotubes and adipocytes, the ratio of myofibrils and fat, hardness, gumminess, resilience, springiness, water content, etc., were similar to those of native fish muscle tissue, but the chewiness was low, and water distribution was different from that of native fish muscle tissue.

In summary, we have successfully developed a pipeline for the production of tissue-like cultured fish fillets. This will be an advanced protocol for different fish and even other economic animal species. It demonstrates that biomimetic scaffolds based on real tissue structure have a great potential in the production of tissue-like cultured meats. For large scale bioprinting, it is difficult to lift spray flow rate and size of extrusion-based 3D printers, due to the limitation of material properties. Simultaneous extrusion of multiple-nozzles will be better choice for large scale tissue culture meat production in the next step.

## Methods

### Isolation and culture of PSCs and PADSCs

The live large yellow croaker was bought from the local seafood market (Hangzhou, Zhejiang, China). The whole fish was soaked in 0.1‰ sodium hypochlorite for half an hour for body surface disinfection, and then anesthetized with 0.1‰ eugenol.

PSCs were isolated and purified as the protocol described in vertebrates^13^. Briefly, the white epaxial muscle was harvested freshly above the lateral line. The muscle was then minced, digested with 0.1% type IV collagenase (Gibco 17104019) for 1 h and 0.1% trypsin-EDTA (Cienry, CR25200) for 20 min successively at room temperature. After filtration with 40μm cell sieve and cell count via hemocytometry, cells were diluted to 1 × 10^6^ cells/ml in high-glucose DMEM (Gibco, C11995500BT) containing 15% FBS (Gibco, 10099141C), 10 ng/ml bFGF (Beyotime, P5453), and 1 × PS (Penicillin-Streptomycin, Macklin, P917928). Finally, the cells were seeded onto PLL (Poly-L-lysine, Sangon, E607015)-coated Petri dishes and cultured at 27 °C with 5% CO_2_.

After removal of the viscera in coelom, adipose tissue was scraped from the abdomen. Then the tissue was treated with 0.1% type I collagenase (Gibco 17100017) solution to vibrate and digest at room temperature for 1 h. After removing the mature adipocytes and passing through the 40μm cell sieve,1 × 10^6^ cells/ml were seeded in high-glucose DMEM containing 15% FBS and 1 × PS in a six-well plate and cultured at 27 °C with 5% CO_2_.

### Cell subculture and differentiation

When the primary cultures grow to 80% cell confluency, a standard subculture process was performed. The 0.25% trypsin-EDTA (Cienry, CR25200) digestive solution was used to resuspend the cells. Passages were performed in the ratio of 1:3 twice a week. And the subcultures were maintained by the DMEM containing 8% FBS, 10 ng/mL bFGF, 1 × PS for PSCs, and DMEM containing 8% FBS and 1 × PS for PADSCs, respectively.

### Myogenic differentiation

PSCs were expanded firstly in complete medium to 80% cell confluency, and then changed to F12 medium (Biosharp, BL311A) containing 8% HS (horse serum, Biosharp, BL209A), 10 ng/mL IGF-1 (Solarbio, P00048), 50 nM necrosulfonamide (Macklin, N872612), 200 μM ascorbic acid (Phytotech, A106), 1×PS, which was renewed half every 3 days.

### Adipogenic differentiation

When PADSCs grew to full confluency, the medium was changed to DMEM/F12 (Gibco, C11330500BT) medium containing 10% HS (horse serum, Biosharp, BL209A), 10 μg/mL insulin (Targetmol, I189675), 0.5 μM IBMX (Targetmol, T1713), 0.25 μM dexamethasone (Targetmol, T0947L), 1% Lipid Mixture (Peprotech, LM-200),1 × PS, to initiate differentiation and renew half of the medium every 3 days.

### High efficiency myogenic differentiation

When PSCs grew to full confluency, the medium was changed to F12 medium containing 8% HS, 5 μM RepSox (Targetmol, T6337), 10 nM LY411575 (Targetmol, T6063), 1 × PS, to initiate high efficiency myogenic differentiation. After 48 h, the same volume of DMEM medium containing 8% FBS, 200 nM dexamethasone and 1 × PS, was added to ensure the survival and continued differentiation of the myotube.

### Immunofluorescent and histological staining

Immunostaining was performed by a general protocol. Cells were fixed with 4% PFA (paraformaldehyde) at RT for 10 min, permeabilized with 0.3% Triton X-100 at RT for 10 min, blocked in blocking buffer (PBS containing 5% goat serum, 1% BSA and 0.3% Tween-20) for 60 min, incubated with the 1st antibody in blocking buffer at RT for 2 h or at 4 °C overnight, and finally counter-stained with 1 μg/ml Hoechst 33342 (BBI, E607328). Pax7 (Bioss, bs-22741R, dilution 1:200), MyoD1 (Abcam, ab209976, dilution 1:200) antibodies for PSCs/myoblast, Desmin (Bioss, bs-1026R, dilution 1:200) for myofiber/myotube, and HoxC9 (Bioss, bs-7982R) for adipocytes were used as 1st antibodies. Goat anti-rabbit IgG secondary antibody, Alexa Fluor 488 (Beyotime, A0423, polyclonal, dilution 1:500) were used as 2nd antibody. All fluorescence images were taken by Olympus FV3000 confocal microscopy.

Nuclei counts were determined by quantifying Hoechst 33342 staining using ImageJ, and normalized against respective controls. The fusion index was determined by quantifying nuclei within desmin-stained myofibres as a proportion of total nuclei and multiplying by 100.

To visualize lipid droplet in adipocytes, after fixation with 4% PFA at RT for 30 min, 3 times PBS washing for 5 min, and soak in 60% isopropyl alcohol for 2 min, Oil red O and hematoxylin staining were performed successively.

### Live/dead staining

The cultured PSCs were stained with 2 μM Calcein-AM and 4 μM PI (Solarbio, CA1630). The cells were thoroughly washed twice with PBS, stained with Calcein-AM for 1 h, then stained with PI for 5 min, washed with PBS for 30 min, and observed under fluorescence microscope (Olympus FV3000) at a wavelength of 490 ± 10 nm. Live cells are green, dead cells are red. Cell viability was calculated as the number of viable cells divided by the total number of cells and counted using ImageJ software.

### RNA sequencing and transcriptome analysis

Sequencing libraries were prepared from harvested RNA samples using the Illumina Truseq^TM^ RNA sample prep Kit and sequenced on the Illumina Novaseq 6000 sequencing platform. Three replicates were performed for each sample. RNA-seq raw data was processed with Fastp (v 0.19.5). 5.4 × 10^7^ (SD = 2.1 × 10^7^) clean reads were obtained from per sample on average.

TopHat (v 2.1.1) was used to align reads to NCBI reference genome L_crocea_2.0 (GCA_000972845.2). 90.1% (SD = 0.36%) of clean reads were uniquely assigned to reference genome. After alignment, transcripts were assembled using Cufflinks (v 2.2.1). To annotate the gene functions, the integrated gene set was aligned against public databases, including NR, Swiss-Port, Pfam, EggNOG, GO and KEGG with BLAST+(v 2.9.0). Gene expression was measured using RSEM (v 1.3.3) with expression values normalized into transcripts per million (TPM).

DEseq2 (v 1.24.0) was used for differential expression analysis. When the log_2_FC cutoff value above 1 and *P*-adjust <0.05, genes were considered differentially expressed and visualized in the volcano map. A heatmap showing the *z* value of all differentially expressed genes between day 0, day 3 and day 6 was constructed in which genes and sample were clustered using Average linkage clustering with Euclidean Distances.

GO enrichment of the selective gene set was performed using Goatools (v 0.6.5). KEGG enrichment of the selective gene set was performed using KOBAS (v 2.1.1).

### 3D cell culture

Appropriate amount of fish gelatin (Yuanye, S25295, 10% m/v), pig gelatin (Sigma, V900863, 10% m/v), silk fibroin (Aladdin, W293432, 15% m/v), hyaluronic acid (Aladdin, H131007, 10% m/v), and chitosan (Aladdin, C105799, 10% m/v) were respectively added to medium. Then, sodium alginate (Aladdin, S100126, 1%) was added to the solution, and the solution was stirred at 70 °C for 3 h to prepare a hydrogel precursor solution. The PSCs (5 × 10^6^ cells/mL) was mixed with each hydrogel precursor solution, and 200 μL was added to a 24-well plate. After solidification with CaCl_2_ solution, 1 mL medium was added and placed in an incubator for cultivation. The medium was changed every two days during the culture. To improve cell viability in 3D culture, 200 μM Pifithrin-α hydrobromide (a p53 inhibitor, Targetmol, T2707,) and 0.1 μM XMU-MP-1a (a Yap activator, Targetmol, T4212) were added to the proliferation medium.

### Rheology measurement

The rheological properties of different bio-inks were evaluated by a rheometer (MCR302, Anton Paar, Austria) equipped with a Peltier element for temperature control. A plate−plate geometry with a diameter of 50 mm was used in all measurements. At first, all hydrogel samples were placed on the plate at 40 °C to completely fill the gap (size of 1 mm) between two plates. The measurements of viscosity and shear stress were performed by varying the shear rate from 1 to 500 s^−1^ with arotational test at 25 °C, respectively. Then, the viscosity was also measured with a temperature ramping from 15 to 40 °C at the rate of 1 °C/min, and the shear rate was maintained at 1 s^−1^. Storagemodulus (G’) and loss modulus (G”) were measured as a function oftemperature at a constant frequency of 1 Hz and a constant strain of 0.1%.

### Micro-CT scanning

As Fish tissue as soft natural biomaterials cannot provide enough X-ray attenuation for micro-CT imaging, a staining method was conducted according to Jeffery et al.^41^ with a slight modification. Sample was cut from the dorsal region of large yellow croaker and fixed in 4% aqueous buffered formaldehyde at room temperature for 24 h, and the fish fillet was stored at −20 °C. Then fish sample was washed with sterile normal saline and weighted and stained by 3.75% IKI solution for 7 d. A high-resolution X-ray microanalyzer (SkyScan1272, Bruker) was utilized for the micro-CT scanning of stained fish tissue. Micro-CT settings were applied as 100 kV and 100 µA, with a relatively low exposure time (1050 ms). A 180° scan was performed with a rotation step of 0.4°, and a relatively high magnification (10 µm/pixel) was used. After the reconstruction of raw data acquiring from rotation steps, axial picture cross sections were provided and then were converted to a 16-bit pictures with a resolution of 1632 × 1632 pixels by cone-beam reconstruction. The 3D visualization and reconstruction software CTvox (Bruker, Germany) in conjunction with Avizo 2019.1 (Thermo Fisher Scientific, United States) were used for analyses.

### 3D bioprinting

A custom-made 3D syringe pump extrusion system was used to print bio-inks. The printing system is located in an ultra-clean room and all components involved, such as syringes, nozzles, and support plates used for cell printing, were sterilized with 70% ethanol and UV treatment. The system consisted of a computer-controlled three-axis positioning stage with automatic movements in the x, y, or z axis for controlling the motion of nozzle and the receiving platform, which had a working space of 45 × 40 × 40 cm^3^ and a syringe pump for extruding the bio-inks. The bio-inks were loaded in a 2 mL syringe equipped with a 400 μm inner diameter conical needle. After printing the muscle scaffold model, it was placed in a 1% CaCl_2_ solution to solidify for 10 min. After rinsing the scaffolds twice with PBS, they were placed in culture medium for 10 days of proliferation and 7 days of differentiation.

### Scanning electron microscope (SEM)

Gel complexes were fixed with glutaraldehyde, followed by dehydration of the complexes with a gradient series of alcohol (30, 50, 70, 80, 90, 95, and 100%), and then dried in a freeze dryer for 24 h. The freeze-dried scaffold was smeared on conductive tape, and excess powder was blown off with an ear wash ball. After coating the scaffold with gold, the morphology of the muscle fibers was observed by SEM (Quanta Feg 250; Hillsboro, USA).

### Tissue slice staining

Paraffin section was made to observe the structure of undyed muscle structure of large yellow croaker. After fish tissue was fixed in 4% formaldehyde at room temperature for 24 h, it was trimmed in the ventilation cupboard with a scalpel and put into the dehydrator (Donatello, DIAPATH, Italy) to dehydrate with gradient alcohol (70–100%, v/v). The tissues were immersed in xylene for transparency and wax was used to soak the sample, ensuring fixation of the fish tissue. Then the wax-soaked tissue was embedded in the embedding machine (JB-P5, Junjie, China), and sliced using a Pathology slicer (RM2016, Leica). Slices were subjected to hematoxylin-eosin and Oil red O, followed by the observation of light microscope (UB200i, UOP, China). The immunostained (Desmin/DAPI) sections were observed with fluorescence microscope, and ImageJ software was used to count the number of nuclei in serial sections to calculate the total number of cells in native fish fillet.

### Textural analysis

The textual properties of empty scaffolds, the scaffolds seeded with PCSs/PADSCs and native fish fillet were measured by a texture analyzer (Universal TA, Shanghai Tengba Instrument Co., Ltd., Shanghai, China) with a flat-surface cylindrical probe (36 mm diameter). The samples were formated strip dimension as 20 mm × 12 mm × 4 mm. The analysis conditions were described as a previous literature^42^ with some modifications, which were set to two compression cycles in the built-in program and held 5 s. The speed was constantly 1 mm/s, while triggering a force of 10 g, and compression height was 30% of the original. The elastic modulus, hardness, springiness, chewiness, gumminess, adhesiveness and resilience were analyzed with Tadviser (Horn Instruments Co., Ltd, Taiwan). The calculated textual properties of experimental samples were compared with the native large yellow croaker for radar chart. The measurements were carried out at room temperature in at least three replicates.

### Low field-nuclear magnetic resonance (LF-NMR)

The LF-NMR analysis of samples were characterized by a previous method^43^ with slight modifications. The relaxation times (T_2_) of the food inks were determined using a nuclear magnetic resonance analyzer (NMI20-015V-I, Niumag Co., Ltd., China). In brief, 5 g samples were weighed and then packed with a thin layer of plastic film. The samples were then placed in 40 mm glass tube before being inserted in the analyzer. Spin–spin relaxation time T_2_ was determined by Carr–Purcell–Meiboom–Gill (CPMG) sequences with the following parameters: time waiting (T_W_) = 3000 ms, time echo (TE) = 0.17 ms, number of echo (NECH) = 18,000, and number of scan (NS) = 4. The data were analyzed by multi-exponential fitting and mono-exponential fitting of T_2_ relaxation data using the software MultiExp Inv Analysis 4.09 (Niumag Electric Co., Ltd., China). The measurements were carried out in three replicates.

### Ethical statement

The large yellow croaker used in this study is an edible economic animal and was purchased from Hangzhou seafood market. After euthanasia, muscle and fat were obtained for experiments. Ethical approval was not required.

## Supplementary information

Supplementary Information

## Data Availability

RNA-seq data has been deposited to the GEO (accession number GSE212904). The authors declare that all data supporting the findings of this study are available in the paper and supplementary information.
