# Supplementary material for: Tissue-like cultured fish fillets through a synthetic food pipeline
Source: NPJ Sci Food. 2023 May 6;7:17. doi: 10.1038/s41538-023-00194-2 (PMC10164169; doi:10.1038/s41538-023-00194-2)
Supplement: Supplementary file 1 — Supplementary Information [file 41538_2023_194_MOESM1_ESM.pdf]

Supplementary Information:

Tissue-like cultured fish fillets through a synthetic food pipeline

Enbo Xu[1,4,5#], Ruihao Niu[1,4#], Jihui Lao[2#], Shengliang Zhang[1,4#], Jie Li[1,4], Yiyuan Zhu[1,4], Huimin Shi[3,5], Qingqing Zhu[1,4], Yijian Chen[2], Yuyan Jiang[2], Wenjun Wang[1,4], Jun Yin[3,4,5], Qihe Chen[1,4], Xiao Huang[2,8]*, Jun Chen[2,4,6,7]*, Donghong Liu[1,4,5]*

[1]College of Biosystems Engineering and Food Science, National-Local Joint Engineering Laboratory of Intelligent Food Technology and Equipment, Zhejiang Key Laboratory for Agro-Food Processing, Integrated Research Base of Southern Fruit and Vegetable Preservation Technology, Fuli Institute of Food Science, Zhejiang University, Hangzhou, 310058, China

[2]College of Life Sciences, Zhejiang University, Hangzhou 310058, China

[3]Key Laboratory of 3D Printing Process and Equipment of Zhejiang Province, School of Mechanical Engineering, Zhejiang University, Hangzhou 310028, China

[4]Innovation Center of Yangtze River Delta, Zhejiang University, Jiaxing 314102, China

[5]State Key Laboratory of Fluid Power and Mechatronic Systems, Zhejiang University, Hangzhou, 310058, China

[6]MOE Key Laboratory of Biosystems Homeostasis & Protection and Innovation Center for Cell Signaling Network, Zhejiang University, Hangzhou, China

[7]Cancer Center, Zhejiang University, Hangzhou, China

[8]Key Laboratory for Cell and Gene Engineering of Zhejiang Province, Department of Ophthalmology, Sir Run Run Shaw Hospital, Zhejiang University School of Medicine, Key Laboratory for Corneal Diseases Research of Zhejiang Province, Hangzhou 310058, China

[#]These authors contributed equally.

*Corresponding author, E-mail address: dhliu@zju.edu.cn; chenjun2009@zju.edu.cn; xh220@zju.edu.cn

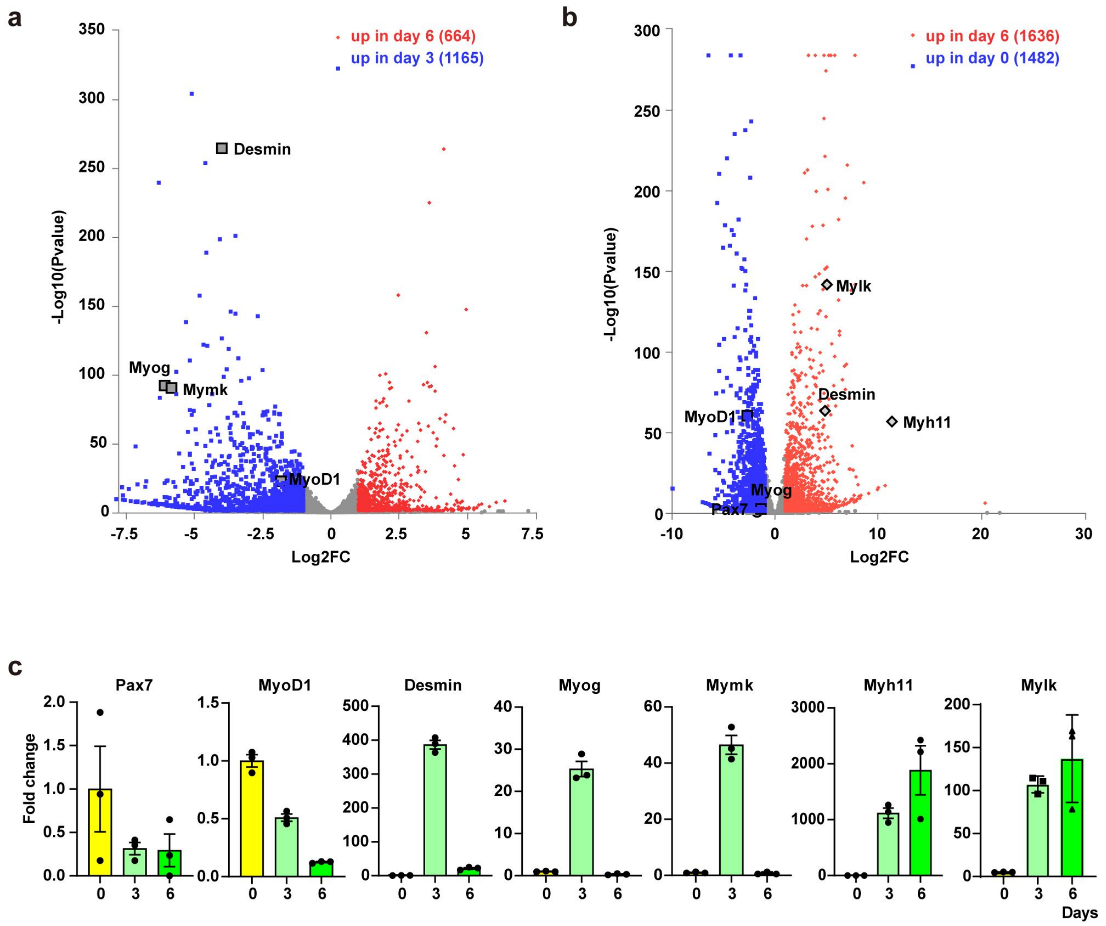

Supplementary Figure 1. Differential gene expression analysis in the myogenic differentiation of PSCs.

a, Volcano plot showing differentially expressed genes between day 3 and day 6. Significantly differentially expressed myogenesis genes were highlighted. **b**, Volcano plot showing differentially expressed genes between day 0 and day 6. Significantly differentially expressed myogenesis genes were highlighted. **c**, The fold change of significantly differentially expressed myogenesis genes from RNA-seq. Error bars for three replicates indicated s.d..

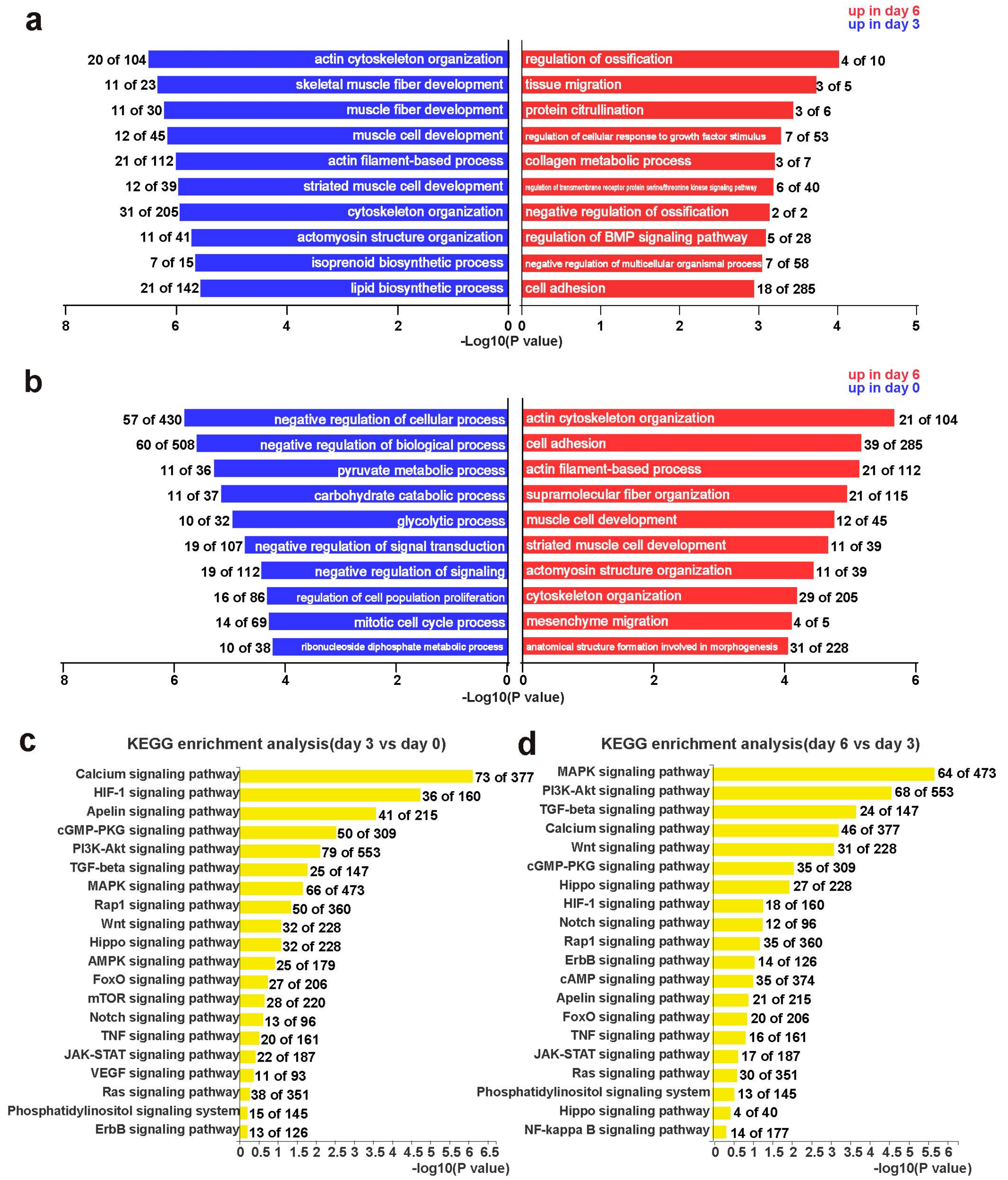

Supplementary Figure 2. GO/KEGG enrichment analysis.

a, Enrichment GO analysis for up-regulation (red bar) and down-regulated (blue bar) DEGs at day 6, compared to day 3. Numbers indicated the proportion of the significantly up/downregulated genes of total genes per gene ontology. **b**, Enrichment GO analysis for up-regulation (red bar) and down-regulated (blue bar) DEGs at day 6, compared to day 0. Numbers indicated the proportion of the significantly up/downregulated genes of total genes

per gene ontology. **c**, Enrichment KEGG analysis for differentially expressed genes corresponding to different signal transduction pathways between day 0 and day 3. Numbers indicated the proportion of the differentially expressed genes of total genes per signal transduction pathway. **d**, Enrichment KEGG analysis for differentially expressed genes corresponding to different signal transduction pathways between day 3 and day 6. Numbers indicated the proportion of the differentially expressed genes of total genes per signal transduction pathway.

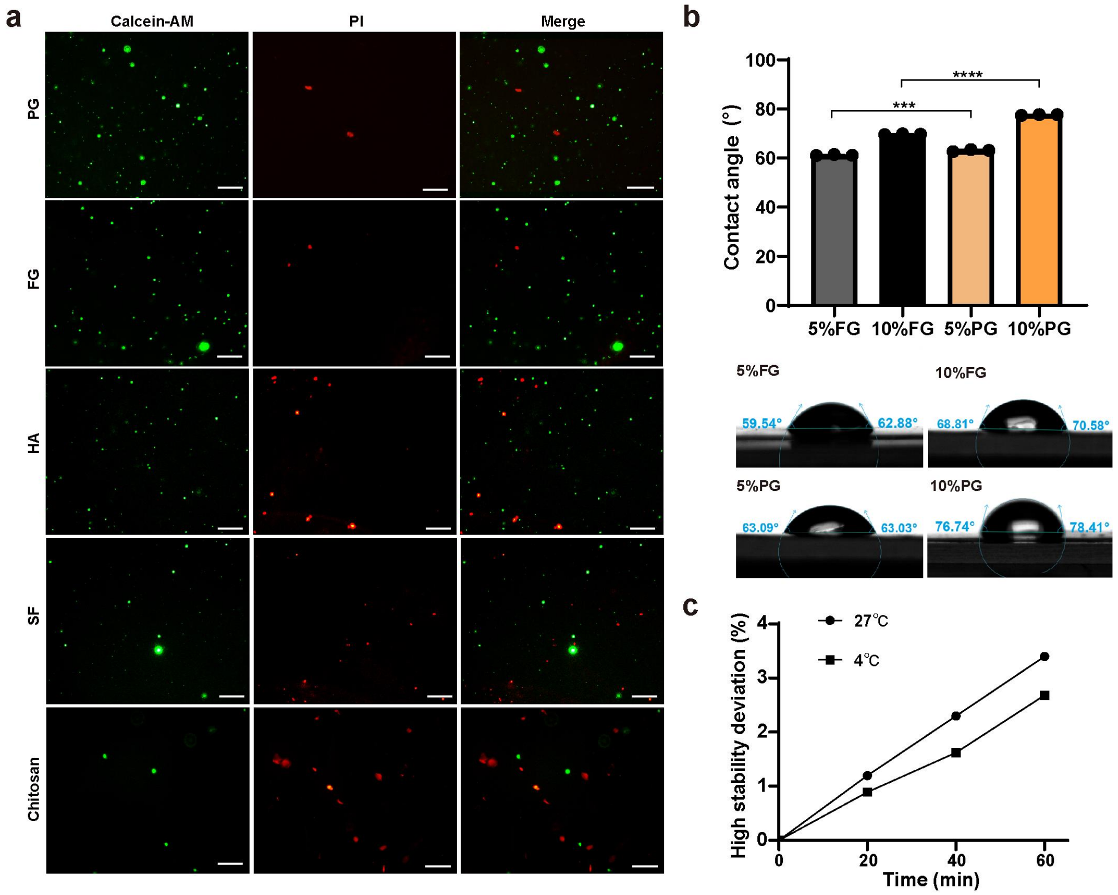

Supplementary Figure 3. Biocompatibility analysis of different scaffold materials for PSCs.

a, Representative fluorescence images of PSCs cultured with different scaffold materials for 5 days. Green: Calcein-AM staining for live cells; Red: PI for dead cells; Scale bar, 50 μm. **b**, Contact angle with different scaffold materials. **c**, Deviation of the height of the printed product.

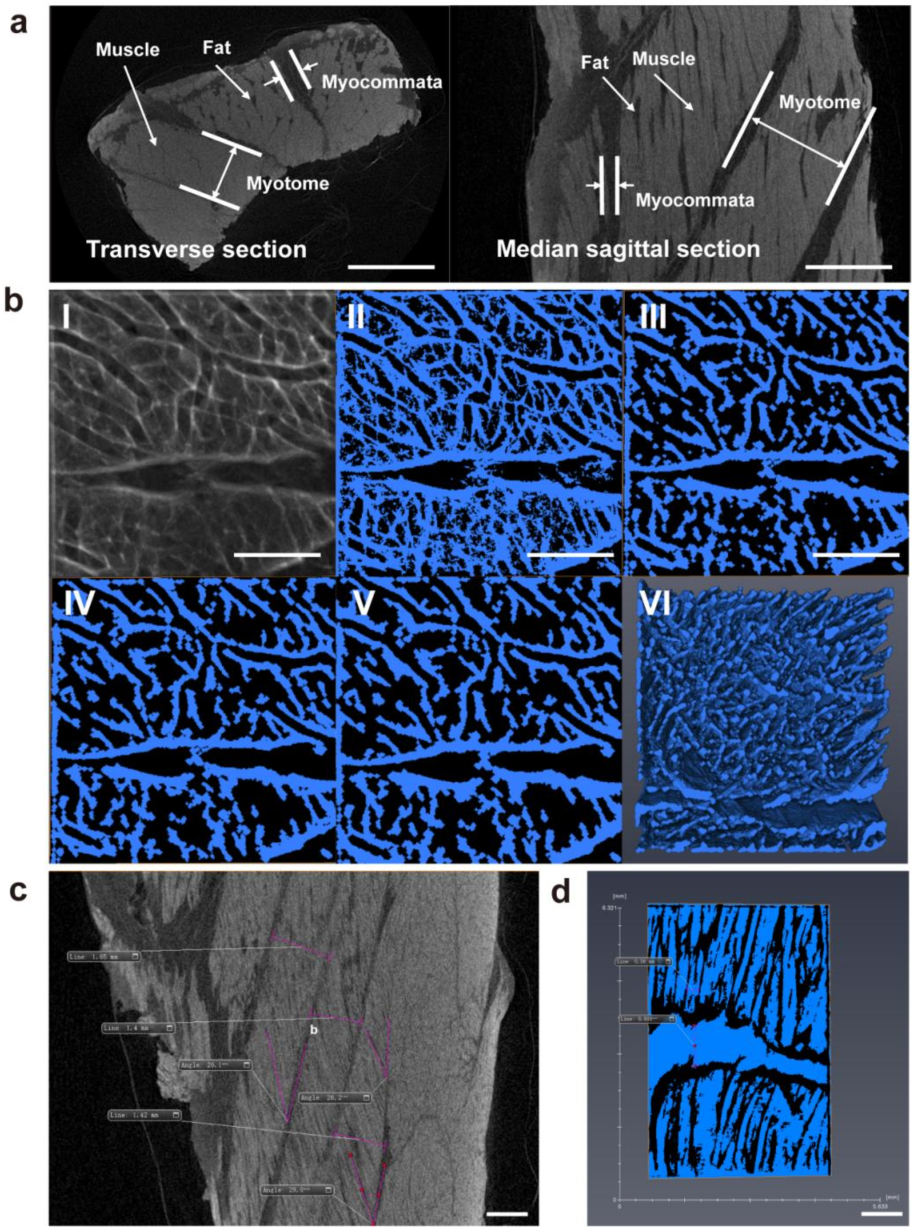

Supplementary Figure 4. Micro-CT reconstruction of fish intermuscular filler model.

a, Transverse section and median sagittal section of micro-CT raw data (epaxial tissue of large yellow cracker). Scale bar, 5 mm. **b**, Flow of denoising and rendered model reconstruction. (I) Micro-CT raw data, (II) Anti-muscle data selection, (III-V) Denoising processing, and (VI) 3D volume rendering. Scale bar, 1 mm. **c**, The width of the myotome and the angle between the muscle fiber and the myosepta were calculated based on the micro-CT raw data. Scale bar, 5 mm. **d**, The width of the myocommata was calculated based on the CT image after threshold segmentation. Scale bar, 1 mm.

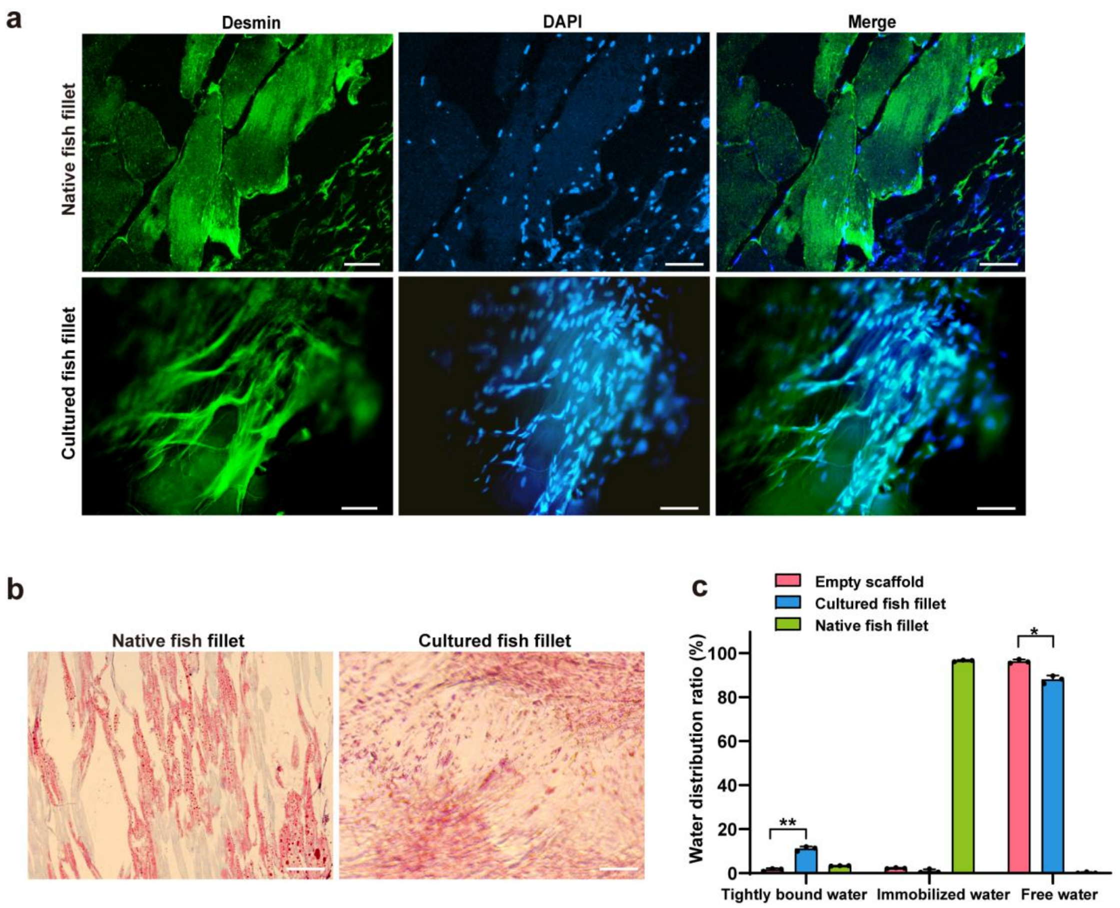

Supplementary Figure 5. Confirmation of myofibers and adipocytes in cell cultured fish fillet.

a, Representative fluorescent stained images of cell cultured fish fillet and native fish fillet. Green: Desmin; Blue: DAPI; Scale bar, 50 μm. **b**, Representative images of cultured fish fillet and native fish fillet with oil red o staining. Red: lipid droplets; Scale bar, 50 μm. **c**, The peak area ratio of T_{2b}, T_{22}, T_{23} in the multi-exponential distribution results was calculated to obtain the tightly bound water, immobilized water, and free water content of each sample.

Supplementary Table 1. Printing parameters

Parameter	Value
GelMA concentration (wt.%)	5-10
SA concentration (wt.%)	1
$CaCl_2$ concentration (wt.%)	0.1-5
Temperature (℃)	24
Print product size (mm)	20 × 12 × 4
Number of repeats	10
Extrusion flow (mL/min)	25
XY moving speed (mm/min)	1200-1500
Conical needle nozzle inner diameter (μm)	400

Supplementary Table 2. Printability analysis

Air pressure (Mpa)	Viscosity at different temperatures			
	15°C 29.8 Pa.s	20°C 11.1 Pa.s	25°C 0.13 Pa.s	30°C 0.03 Pa.s
0.1	unextrudable	unextrudable	unextrudable	irregular
0.2	unextrudable	unextrudable	printable	irregular
0.3	unextrudable	unextrudable	printable	irregular
0.4	unextrudable	irregular	printable	irregular
